# Current Status and Challenges of Support Environments for New Graduate Occupational Therapists in Japanese Hospitals: A Mixed Method Study

**DOI:** 10.1155/2022/2159828

**Published:** 2022-09-05

**Authors:** Shigehito Shiota, Naoya Goto, Aki Kanayama, Yuko Kubo, Kazuhiko Hirata, Yoshihiro Ito, Hiroaki Kimura

**Affiliations:** ^1^Department of Rehabilitation, Division of Clinical Support, Hiroshima University Hospital, Hiroshima, Japan; ^2^Division of Clinical Support, Hiroshima University Hospital, Hiroshima, Japan; ^3^Department of Rehabilitation, Hiroshima University Hospital, Hiroshima, Japan

## Abstract

**Introduction:**

The number of occupational therapists in Japan continues to increase, which has led to an urgent need to improve the educational environment for new graduate occupational therapists. This study attempted to identify the current state and challenges of the educational environment for new graduate occupational therapists in Japan.

**Methods:**

We employed a mixed method using quantitative and qualitative data from a questionnaire survey of 1055 chief occupational therapists in Japanese hospitals. The questionnaire consisted of (1) basic information about the respondent and their hospital, (2) educational environment for new graduate occupational therapists at the respondent's hospital, (3) time spent on in-hospital lectures and on-the-job training, and (4) challenges in the clinical education of new graduate occupational therapists. Text data was analysed qualitatively using text mining to create cooccurrence networks.

**Results:**

A total of 385 responses were obtained with a response rate of 36.5%. All hospitals had recruited new graduate occupational therapists in the last five years, but more than half of them did not have a philosophy and policy on education and had not prepared guidelines for the completion of occupational therapy education. The cooccurring network of issues in the educational environment for new graduate occupational therapists indicates the need to create standard guidelines, train supervisors, and develop a hospital education system.

**Conclusion:**

In the future, the needs of the educational environment for newly graduated occupational therapists should be investigated, and standardised educational guidelines should be developed.

## 1. Introduction

Japan has the fastest aging population in the world, surpassing 360,000 in 2020, which accounts for 28.7% of the population [1]. As a high proportion of the elderly requires rehabilitation and care, the need for occupational therapy is increasing to maintain their health and improve their quality of life. As this need increases, the number of occupational therapists in Japan continues to grow, exceeding 100,000 in 2020, giving Japan the second largest number of occupational therapists in the world [2, 3]. Additionally, approximately 5,000 new graduate occupational therapists transition from student to clinical therapist each year [4]. Occupational therapists in Japan are characterised by young age, with around a quarter (27.5%) having less than five years of clinical experience and 73.2% working in hospitals [5].

In recent years, as healthcare has become more sophisticated and diverse, healthcare professionals are required to have higher levels of expertise and skill. Occupational therapists working in hospitals have a duty to provide safe, high-quality occupational therapy to clients, and new graduate occupational therapists transitioning from studying to clinical practice need to continue their education [6]. In addition, new graduate occupational therapists have been identified as being at risk of turnover and burnout due to the ethical tensions they face, and it is important to have a system in place where they can access support [7, 8]. The provision of an appropriate educational environment for new graduate occupational therapists and the presence of a mentor will facilitate their adaptation to the workplace and prevent disengagement [9]. There are scattered reports on mentoring for occupational therapy students and researchers, but few studies for clinicians [10, 11]. To improve the satisfaction of new graduate occupational therapists and clients, hospitals need to provide a consistent educational environment for the former, from entry-level education to continuing professional education. However, it is unclear what type of educational environment is provided for new graduate occupational therapists in hospitals. In order for clients and new graduate occupational therapists to feel comfortable practicing occupational therapy, the current state of education in hospitals needs to be improved.

The aim of this study was to identify the current situation and issues related to the educational environment for newly graduated occupational therapists through a questionnaire survey of hospitals in Japan. By clarifying the current state of the environment and issues in the education of new graduate occupational therapists, this study is expected to help inform the development of a standardised education programme for new graduate occupational therapists in clinical practice.

This paper defines a new graduate occupational therapist as an occupational therapist within one year of transitioning from studying to clinical practice. A “supervisor” is an experienced occupational therapist whose role is to supervise the provision of occupational therapy programmes to help newly graduated occupational therapists develop, improve, and maintain patients' abilities to perform daily activities.

## 2. Materials and Methods

### 2.1. Study Design

**Figure 1:**
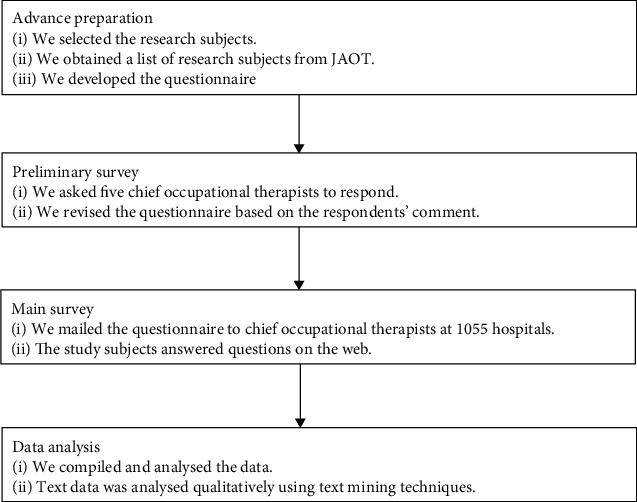
Overview of survey development and implementation process.

We adopted a mixed method approach from the questionnaire survey, combining quantitative and qualitative methods. We used text mining methods to analyse the qualitative data. Text mining methods are a means of transforming text data into sets of words, combining them with structured data, and extracting information by looking at their correlations, biases, and time series [12–14].  Figure 1 shows an overview of the research process.

### 2.2. Participants

We included 1055 chief occupational therapists from hospitals with at least ten members of the Japanese Association of Occupational Therapists (JAOT) in our study population. We used the JAOT member portal to select study participants. Previous studies of physiotherapists reported that hospitals with more than ten physiotherapists had a good educational environment for new graduates [15]. Therefore, we decided to target this study at chief occupational therapists in hospitals with at least ten occupational therapists. Contact information of the participants was obtained by submitting a written pledge of confidentiality to the JAOT. In addition, after obtaining the approval of the executive director of the JAOT, who was responsible for the management of personal information of the JAOT, we obtained address stickers with the addresses and names of the facilities.

### 2.3. Data Collection and Measures

#### 2.3.1. Preparation of the Questionnaire

We developed a questionnaire based on previous research questionnaires [15]. In addition, we asked an open-ended question about the challenges of supporting new graduate occupational therapists. As a preliminary study, we emailed a questionnaire to five chief occupational therapists working in Japanese hospitals, obtaining responses and comments. The questionnaire was revised based on the feedback comments.

The survey comprised a total of 17 questions divided into four categories (Supplementary material (available here)). The questionnaire is a combination of multiple choice and open-ended questions, and the survey questions are as follows: (1) basic information about the respondents and their hospitals, (2) educational environment for new graduate occupational therapists in respondents' hospitals, (3) time spent on in-hospital lectures and on-the-job training, and (4) challenges in the clinical education of new graduate occupational therapists. We created forms using Google Forms, so the completed questionnaires could be answered on the web.

#### 2.3.2. Main Survey

The questionnaire-based survey was conducted from 15 February to 22 March 2019. The questionnaire was mailed to the chief occupational therapists at the participating facilities, with an explanatory document explaining the purpose and objectives of the study. The explanatory materials included survey URLs and QR codes, which allowed participants to answer the survey not only on computers but also on smartphones and tablet devices.

### 2.4. Statistical Analysis

We calculated a percentage score after a simple tabulation. Free-text descriptions were analysed using text mining methods and KH Corder (Ver. 3. Aloha 1.7k), a software for quantitative content analysis for Japanese texts [12]. Frequent words were calculated, and cooccurrence networks were created using the text mining data. Cooccurrence network diagrams present words that have a similar pattern of occurrence among the frequent words in the text data. Therefore, the network diagram consists of lines connecting words with strong cooccurrence, with the stronger the cooccurrence relationship, the thicker the line, and the larger the circle for words with high frequency of occurrence.

### 2.5. Ethical Considerations

This study was conducted in accordance with the principles of the Declaration of Helsinki. We obtained approval from the Hiroshima University of Epidemiological Research Ethics Review Board (Approval No.: E–1339). An explanatory document, which was mailed along with the questionnaire, informed participants of the purpose and methods of the study, ethical considerations, and that participation in the study was voluntary and their refusal to participate would have no consequences. Participants were considered to have consented to the study by answering the questionnaire.

## 3. Results

We received responses from the 385 chief occupational therapists, resulting in a response rate of 36.5%.  Table 1 shows basic information on respondents and their hospitals. Nearly a third (30.9%) of respondents had the most years of experience as an occupational therapist (11-15 years), followed by 24.9% (16-20 years) and 17.9% (21 years or more). General hospitals were the most common hospital to which respondents belonged (87.2%). More than half (58.7%) of respondents had 10-20 occupational therapists in their hospitals, but more than 10% had 41 or more. All hospitals where respondents belonged had employed at least one new graduate occupational therapist within the last five years.

The results of the educational environment for new graduate occupational therapists show that most hospitals had prepared an education plan and supervisor, but less than half had a philosophy or policy in place (Table 2). Furthermore, more than 90% of the respondents stated that the assignment of new graduate occupational therapists to supervisors was in the ratio of 1 : 1. The most common number of years of clinical experience required of supervisors as occupational therapists was 3 years (29.9%), followed by 2 years (27.3%) and 5 years (19.5%). About half of the criteria for completing education for new graduate occupational therapists was completion of their hospital's own education programme, while 40% of the theories had no criteria. Few hospitals (2.6%) used the JAOT Post-Qualification Education System as a completion criterion for new graduate occupational therapists.

Table 3 shows the time spent teaching in in-hospital lecture sessions and on-the-job training (OJT) in clinical practice. In lecture sessions, about 25% of the hospitals spent more than 20 hours on occupational therapists' professional knowledge and skills, while less time was spent on hospitality, working skills education, and research methods. The OJT in clinical settings revealed that 42.3% of hospitals have new graduate occupational therapists that spend more than 20 hours in supervisory observation. In addition, in more than half of these hospitals, supervisors spent 1-2 hours reviewing new graduate occupational therapists' medical records and reports and conducting one-on-one meetings with them.

**Figure 2:**
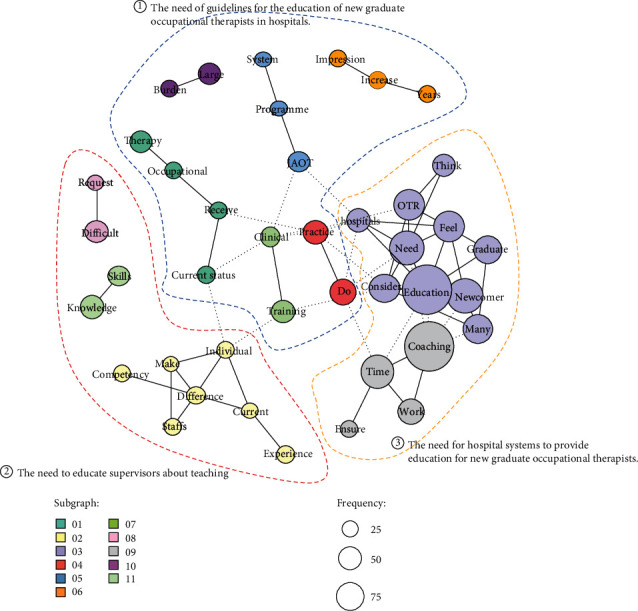
Cooccurring networks of issues in the education of new graduate occupational therapists. (*n* = 144).

Of the 385 total respondents, we received 144 responses on “the challenges in the clinical education for new graduate occupational therapists (open-ended).” The results of cooccurrence networks using text mining are shown in Figure 2. We extracted the top 41 most frequent words in the textual data and created a cooccurrence network. The text was divided into 11 subcategories, and we further divided these subcategories into three categories: (1) the need for guidelines for the education of new graduate occupational therapists in hospitals, (2) the need to educate supervisors about teaching, and (3) the need for hospital systems to provide education for new graduate occupational therapists.

## 4. Discussion

In this study, we have identified the current situation and challenges in the educational environment for new graduate occupational therapists through a questionnaire survey of hospitals across the country. Although this survey was distributed to many participants (1055), the response rate was only 36.5%, which was similar to the response rate of 37.7% in previous studies [15]. Based on the results of our cooccurrence network, we identified three needs as challenges in the educational environment for new graduate occupational therapists. The following section discusses these three areas.

### 4.1. The Need for Guidelines for the Education of New Graduate Occupational Therapists in Hospitals

All hospitals of the respondents to this survey had employed new graduate occupational therapists in the last five years. However, we found that 53.2% of hospitals had no philosophy or policy on education, 37.9% had no chart to assess the skills of new graduate occupational therapists, and 39.0% had no criteria for the completion of education for new graduate occupational therapists. Philosophy and policy point the way forward for the organisation and are important elements in the education of occupational therapists. Therefore, it is necessary to develop a philosophy and policy regarding how each hospital wants new graduate occupational therapists to develop. One of the outcomes of occupational therapy education in hospitals is an assessment of the clinical skills of new graduate occupational therapists. The World Federation of Occupational Therapists (WFOT) has published the Quality Evaluation Strategy Tool (QUEST) as an occupational therapy quality measure [16]. Several countries have indicated competencies for entry-level occupational therapists tailored to their respective cultures and systems (Australia: Australian Occupational Therapy Competency Standards (AOTCS) [17], Canada: The Association of Canadian Occupational Therapy Regulatory Organization (ACOTRO) Essential Competencies [18], Europe: European Network of Occupational Therapy and Higher Education (ENOTHE) [19], United Kingdom: Health and Care Professions Council (HCPC) [20], and United States of America: Standards of Practice for Occupational Therapy) [21]. In addition, several disease-specific occupational therapy skills competencies have been developed [22, 23]. We believe that these indicators and competencies should be introduced as an assessment of the level of skill acquisition of new graduate occupational therapists and guidelines should be developed. Most hospitals did not utilise the completion of the JAOT Post-Qualification Education System for the completion of education for new graduate occupational therapists. The JAOT Post-Qualification Education System includes the Post-qualification Basic Training Programme to support occupational therapists' continuous self-improvement; the Certified Occupational Therapist System to acquire certain skills in occupational therapy, such as clinical, educational, research, and management; and “the Specialist Occupational Therapist System” to acquire advanced professional skills related to occupational therapy [24]. The lack of common standards makes it difficult to compare the quality of the educational environment for new graduate occupational therapists. In Japan, the Japanese Nursing Association has published “guidelines for training new nurses” [25], and the Japanese Association of Physiotherapists provides “guidelines for training new physiotherapy staff” [26]. We believe that JAOT needs to develop guidelines for the education of new graduate occupational therapists that can be commonly used in all hospitals.

### 4.2. The Need to Educate Supervisors about Teaching

Almost all hospitals had one supervisor for newly graduated occupational therapists. On the other hand, more than half of the respondents stated that the supervisor needed two to three years of clinical experience. For occupational therapists in Japan to become supervisors of students' clinical practice, they need five years of clinical experience as occupational therapists and attend a training course [27]. Therefore, it was suggested that in the real clinical environment, young occupational therapists who do not fulfil the requirements for clinical practice supervisors may be supervising newly graduated occupational therapists. The presence of a mentor is reported to be highly associated with high job satisfaction and good clinical fit among new graduate occupational therapists [9]. We believe that educational support for supervisors is also necessary to alleviate the stress of new graduate occupational therapists and to ensure a good adjustment to the workplace. Coaching has recently received attention as an educational tool to improve the nontechnical skills of healthcare professionals, and education of supervisors in coaching techniques may be increase the effectiveness of OJT [28]. The JAOT Post-Qualification Education System includes an “education” curriculum, but it focuses on education for occupational therapy students, with little mention of education for new graduate occupational therapists [24]. The educational environment for supervisors of new graduate occupational therapists also needs to be improved.

### 4.3. The Need for Hospital Systems to Provide Education for New Graduate Occupational Therapists

Conferences on the education of new graduate occupational therapists were held in 70.1% of respondents' hospitals. In addition, many hospitals spent a lot of teaching time on lectures on occupational therapy knowledge and skills and on supervisors' observation of clinical situations. Under the Japanese healthcare system, the reimbursement obtained is determined by the time spent with the patient as one 20-minute unit [29]. Therefore, the more time a hospital spends educating new graduate occupational therapists to provide quality occupational therapy to clients, the less reimbursement the hospital will get. It is very important to balance revenue and education, so it is necessary to develop a system for training new graduate occupational therapists in hospitals. Sandra et al. reported that new graduate occupational therapists felt they lacked technical intervention skills and needed between six months and two years to feel competent [30]. We propose an educational environment that provides new graduate occupational therapists with a two-year intensive programme of education, including coaching skills for transition to the role of supervisor.

The study had several limitations. First, the participants in this study were from hospitals with at least ten JAOT occupational therapists. The status of the educational environment in hospitals with a small number of occupational therapists is unknown and therefore requires ongoing research. Second, the respondents were only chief occupational therapists, which may be a one-sided view of those providing instruction. We need to investigate the preferences of newly graduated occupational therapists for their learning environment. Third, the questionnaire in this study was based on the questionnaire used in the survey of physiotherapists and did not contain sufficient items on occupational therapy. Future surveys with questionnaires including items on occupational therapy are encouraged.

## 5. Conclusion

In this study, we have identified the current situation and challenges in the educational environment for new graduate occupational therapists through a questionnaire survey of chief occupational therapists in 1055 hospitals in Japan. All hospitals had hired new graduate occupational therapists within the last five years, but more than half of them had no philosophy or policy on education and no clear guidelines for completing education. We believe that standardised guidelines for training new graduate occupational therapists, training for supervisors, and development of an educational environment in hospitals are needed.

## Figures and Tables

**Table 1:** Basic information about the respondents and their hospitals (*n* = 385).

	*n*	%
Respondent's years of experience as an occupational therapist		
1–5 years	42	11.0
6–10 years	59	15.3
11–15 years	119	30.9
16–20 years	96	24.9
Over 21 years	69	17.9
Respondent's type of hospital		
University hospitals	10	2.6
General hospitals	335	87.1
Psychiatric hospitals	32	8.3
Clinics	2	0.5
Others	6	1.5
Number of occupational therapists affiliated with the respondent's hospital		
10–20 persons	226	58.7
21–30 persons	89	23.1
31–40 persons	29	7.5
More than 41 persons	41	10.7
Number of new graduate occupational therapists hired by respondent's hospital in the last 5 years		
0	0	0.0
1–5	117	30.4
6–10	113	24.5
11–15	75	19.5
16–20	21	5.6

**Table 2:** Educational environment for new graduate occupational therapists in respondents' hospitals (*n* = 385).

	*n*	%
Philosophy and policy regarding the education of new graduated occupational therapists		
Yes	180	46.8
No	205	53.2
Education plan for new graduated occupational therapists		
Yes	341	88.6
No	44	11.4
Conduct a conference for new graduate occupational therapists		
Yes	270	70.1
No	115	29.9
Assessment chart to evaluate the skills of new graduate occupational therapists		
Yes	239	62.1
No	146	37.9
Supervisor teaching new graduate occupational therapists		
Yes	375	97.4
No	10	2.6
Number of new graduate occupational therapists assigned per supervisor		
1 person	335	87.2
2 persons	30	7.8
Over 3 people	19	4.9
Years of experience as an occupational therapist required for supervisor		
2 years	105	27.3
3 years	115	29.9
4 years	45	11.7
5 years	75	19.5
6–10 years	40	10.4
Over 11 years	5	1.3
Criteria for completion of education for new graduate occupational therapists		
Completion of the hospital's own educational programme for new graduate occupational therapists	173	44.9
Completion of case reports or academic presentations	28	7.3
Completion of the JAOT Post-Qualification Education System	10	2.6
There are no specific criteria	150	39.0
Others	24	6.2

**(a):** 

	*n* (%)				
In-hospital lecture sessions (per year)	1-5 hours	6-10 hours	11-15 hours	16-20 hours	Over 20 hours
General orientation of facilities	256 (66.5)	61 (15.8)	10 (2.6)	16 (4.2)	42 (10.9)
Hospitality and patient care	331 (86.0)	37 (9.6)	7 (1.8)	0 (0.0)	10 (2.6)
Social skills education	328 (85.2)	31 (8.0)	10 (2.6)	5 (1.3)	11 (2.9)
Risk management	292 (75.9)	57 (14.8)	12 (3.1)	6 (1.6)	18 (4.7)
Expertise in occupational therapy	163 (42.3)	74 (19.2)	39 (10.1)	19 (4.9)	90 (23.4)
Professional skills in occupational therapy	174 (45.2)	72 (18.7)	28 (7.3)	17 (4.4)	94 (24.4)
Case presentation	239 (62.1)	53 (13.8)	31 (8.1)	11 (2.8)	51 (13.2)
Research methods	335 (87.0)	21 (5.5)	8 (2.1)	7 (1.8)	14 (3.6)

**(b):** 

On-the-job training (per month)	1–2 hours	6–10 hours	11–15 hours	16–20 hours	Over 20 hours
New graduate occupational therapists observe a supervisor in a clinical practice	115 (29.9)	40 (10.4)	35 (9.1)	32 (8.3)	163 (42.3)
Supervisors review and guide the clinical practice of new graduate occupational therapists	159 (41.3)	66 (17.2)	29 (7.5)	37 (9.6)	94 (24.4)
Supervisors check medical records and reports for guidance.	215 (55.8)	66 (17.1)	33 (8.6)	19 (4.9)	52 (13.5)
One-on-one meetings	248 (64.4)	64 (16.6)	25 (6.5)	11 (2.9)	37 (9.6)

## Data Availability

The data used to support the findings of this study are available from the corresponding author upon request.

## Supplementary Materials

Supplementary Materials The supplementary file is the questionnaire used in this study.
