# Supplementary material for: Current Status and Challenges of Support Environments for New Graduate Occupational Therapists in Japanese Hospitals: A Mixed Method Study
Source: Occup Ther Int. 2022 Sep 5;2022:2159828. doi: 10.1155/2022/2159828 (PMC9467731; doi:10.1155/2022/2159828)
Supplement: Supplementary Materials — The supplementary file is the questionnaire used in this study. [file 2159828.f1.docx]

**Supplemental materials**

**Questionnaire**

“Survey on the current status and Challenges of support environment for new graduate occupational therapists in Japanese hospitals.”

(1) Basic information about the respondents and their hospitals

　①　Respondent's years of experience as an occupational therapist

(please enter the number that applies)

*( 　　　　　　　) year(s) of experience*

　②　Respondent's type of hospital (Please check one item that applies)

- *University hospital　　　　　　　□　General hospital*
- *Psychiatric hospital □　Clinic*
- *Others　（　　　　　　　　　）*

③　Number of occupational therapists affiliated with the respondent's hospital

(please enter the number that applies)

*( ) Number of persons*

　④　Number of new graduate occupational therapists hired by respondent's hospital in the last 5 years (please specify the relevant number)

*(　　　　　　　 ) Number of persons*

(2) Educational environment for new graduate occupational therapists in respondent’s hospitals

　①　Philosophy and policy regarding the education of new graduate occupational therapists (Please check one item that applies)

- *Yes 　　　　　　　□　No*

　②　Education plan for new graduate occupational therapists

(Please check one item that applies)

- *Yes 　　　　　　　□　No*

　③　Conduct a conference for new graduate occupational therapists

(Please check one item that applies)

- *Yes 　　　　　　　□　No*

　④　Assessment chart to evaluate the skills of new graduate occupational therapists

(Please check one item that applies)

- *Yes 　　　　　　　□　No*

⑤　Supervisor teaching new graduate occupational therapists

(Please check one item that applies)

- *Yes 　　　　　　　□　No*

　⑥　Number of new graduate occupational therapists assigned per supervisor

　(Please check one item that applies)

- *1 person　　　　　　□ 2 persons*
- *Over 3 people*

　⑦　Years of experience as an occupational therapist required for supervisor

　(Please check one item that applies)

- *２ Years 　　　　　□ 3 Years*

*□　4 Years □ 5 Years*

*□　6-10 Years □ Over 11 years*

　⑧　Criteria for completion of education for new graduate occupational therapists

(Please tick all that apply)

- Completion of the hospital's own educational programme for new graduate occupational therapists.
- Completion of case reports or academic presentations
- Completion of the JAOT Post-Qualification Education System
- There are no specific criteria
- Others　（　　　　　　　　　　　　　　　　　　　　　　）

(3) Time spent on in-hospital lectures and on-the-job training

　①Please provide the actual hours spent on in-hospital sessions in figures (per year)

| In-hospital lecture sessions | hours/years |
| --- | --- |
| General orientation of facilities |  |
| Hospitality and patient care |  |
| Social skills education |  |
| Risk management |  |
| Expertise in occupational therapy |  |
| Professional skills in occupational therapy |  |
| Case presentation |  |
| Research methods |  |

　②Please provide the actual hours spent on On-the job Training in figures (per month)

| On-the-job training | hours/month |
| --- | --- |
| New graduate occupational therapists observe a supervisor in a clinical practice |  |
| Supervisors review and guide the clinical practice of new graduate occupational therapists |  |
| Supervisors check medical records and reports for guidance. |  |
| One-on-one meetings |  |

(4) Challenges in the clinical education of new graduate occupational therapists.

　（Please state in open-ended in the text.）

|  |
| --- |
